# Thermoluminescent microparticle thermal history sensors

**DOI:** 10.1038/micronano.2016.37

**Published:** 2016-08-26

**Authors:** Joseph J. Talghader, Merlin L. Mah, Eduardo G. Yukihara, Adam C. Coleman

**Affiliations:** 1 Department of Electrical and Computer Engineering, University of Minnesota, Minneapolis, MN 55455, USA; 2 Department of Physics, Oklahoma State University, Stillwater, OK 74078, USA

**Keywords:** materials, microparticles, sensors, thermoluminescence

## Abstract

While there are innumerable devices that measure temperature, the nonvolatile measurement of thermal history is far more difficult, particularly for sensors embedded in extreme environments such as fires and explosions. In this review, an extensive analysis is given of one such technology: thermoluminescent microparticles. These are transparent dielectrics with a large distribution of trap states that can store charge carriers over very long periods of time. In their simplest form, the population of these traps is dictated by an Arrhenius expression, which is highly dependent on temperature. A particle with filled traps that is exposed to high temperatures over a short period of time will preferentially lose carriers in shallow traps. This depopulation leaves a signature on the particle luminescence, which can be used to determine the temperature and time of the thermal event. Particles are prepared—many months in advance of a test, if desired—by exposure to deep ultraviolet, X-ray, beta, or gamma radiation, which fills the traps with charge carriers. Luminescence can be extracted from one or more particles regardless of whether or not they are embedded in debris or other inert materials. Testing and analysis of the method is demonstrated using laboratory experiments with microheaters and high energy explosives in the field. It is shown that the thermoluminescent materials LiF:Mg,Ti, MgB_4_O_7_:Dy,Li, and CaSO_4_:Ce,Tb, among others, provide accurate measurements of temperature in the 200 to 500 °C range in a variety of high-explosive environments.

## Introduction

Temperature measurement devices abound: a simple web search for commercial products using the keywords ‘temperature sensor’ gives almost 1000 different possibilities, and these primarily only encompass general consumer-oriented applications. It is a far different story when one searches for ‘thermal history sensors’. Here there are no immediate commercial products, only scholarly articles. This partially reflects the rarity of technologies that can measure and store temperature and time in some manner without internal or external sources of power. The difficulty is compounded in applications in metallurgy^1,2^, VLSI processing^3,4^ and laser machining^5,6^, to name only a few, when the device must be unobtrusive and robust enough to be incorporated in a wide variety of heat treatments at temperatures that will destroy typical nonvolatile electronics.

Imagine now the difficulty of measuring temperature or thermal history inside an explosion. The environment in and around the fireball is surely one of the harshest environments on Earth, yet bioagents such as anthrax spores may trace complex paths through it to emerge unscathed^7^. While optical sensing techniques such as pyrometry^8^, atomic emission spectroscopy^9,10^, and coherent anti-Stokes Raman scattering^11^ can determine temperature at a distance, these generally measure light emission from the chemical reaction itself rather than the temperature of the blast zone and surrounding objects. Direct contact sensors such as thermocouples that may survive the detonation are fixed in place, dependent on thermally-conductive wires, and too massive to register the full extent of a very rapid temperature excursion.

In all of the above high-temperature applications, if one wishes to assess thermal history representatively as a function of position, then one needs thermal history sensors inside the thermal event, be it, say, a laser annealing process or an explosion. These sensors will need to be small and robust so that they can follow rapid temperature changes without damage, they must be free of internal or external communication or power supplies since these will seldom be compatible with the thermal event, and they must be easily extracted and read without interfering background signals.

Thermoluminescent microparticles have many advantages that make them suitable as harsh environment temperature and thermal history sensors.

1. The particles are tough, cheap, can be produced by the billions, and have no ‘parts’ that can be damaged by thermal events.
2. Particle thermoluminescence (TL) is primed by radiation prior to use (months in advance, if desired), whereas the background materials and debris which will mingle with the particles in the course of use do not receive pre-treatment; thus, virtually all luminescence observed on heating of a recovered sample will come from the particles within. Given this limited noise, only tiny numbers of particles—sometimes only one—are necessary to make a measurement, which can be done while still mixed with contaminants and debris.
3. Variations in the thermal history can be seen from the differences in luminescence from region to region or particle to particle.
4. Multiple particle types can be combined to create arbitrary temperature measurement ranges.

The following pages will be dedicated to explaining and justifying these claims, but there are other thermal history sensing technologies that have been developed in the past and it is to these that we will now turn.

## Thermal history sensing: prior art

When issues of extremely small size, zero power draw, and high temperature are taken into account, the prior art for *in situ* thermal history relies almost exclusively on materials properties that change depending on maximum temperature and the duration over which it is applied. The applications are very diverse and quite interesting. Raffaëlly-Veslin *et al*.^12^ used signatures in the Raman spectra of glass from the ancient Roman period to determine the method by which the glass was manufactured. Cherepanov *et al*.^13^ noted alterations in the Mossbauer spectra of superconducting yttrium barium copper oxide based on the heat treatments that the material received during processing. Fair *et al*.^14^ have used the crystal microstructure of composites of glass ceramics to measure thermal dose. This is an interesting concept where a well-calibrated microstructure pattern can be correlated to the likely microstructure of the material it is monitoring. Changes in the morphology of nanostructures have also been proposed to assess thermal history. Sun *et al*.^15^ have observed that thin islands of gold metal alter in response to thermal dose, forming shapes with reduced surface-to-volume ratios. The group of Huang^16^ has used TiO_2_ nanoparticles as sensors by analyzing changes in their Raman spectra after they have grown during heating. Researchers such as Mecerreyes^17^ and Sukwattanasinitt^18^ have developed irreversible thermochromic materials to record and visually indicate temperature maximums through altered spectral absorbance.

Most of the remaining few thermal history sensors utilize fluorescence and photoluminescence in some form. Commonly this involves a defect in a solid-state crystal or particle, such as Y_2_O_3_:Eu. The emission and absorption characteristics of these defects are very sensitive to the local bonding environment and microstructure of the material and therefore its thermal history. Perhaps the earliest work of this type was that of Feist^19,20^, but subsequent important research has also been performed by Eilers^21^ and Cornu^22^. In these methods, after a thermal event, a laser or optical parametric oscillator is used to excite carriers to the upper defect levels where a subsequent fluorescent event emits photons. Thermal data can be extracted from among many properties, especially the fluorescence peak, its linewidth, or lifetime. Semiconductor core-shell nanoparticles have been used by Chen^23^ in a somewhat similar manner except that in this case, the band gaps of the nanoparticles change as the core and shell materials interdiffuse over time at high temperatures. The shift in the position and shape of the photoluminescence curve is then used to extract information about temperature.

Finally, TL, and by extension, optically stimulated luminescence (OSL) in particles and thin films can be used to monitor thermal history^24–28^. This technique has been shown in extensive field testing to give accurate temperature and time data in explosions and will be discussed in great detail in this paper.

## Thermal history sensing concepts

### Thermoluminescent traps and thermal history sensing

**Figure 1:**
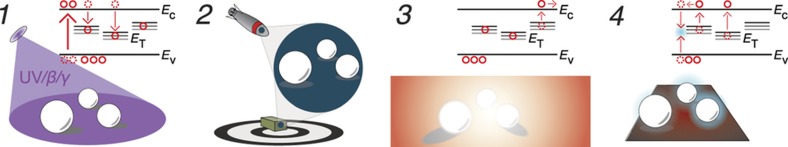
Conceptual diagram of the use of thermoluminescent microparticles to measure the thermal history of the periphery of an explosion. After Ref 27. UV, ultraviolet.

TL and OSL materials have applications in dosimetry^29,30^ and archeological dating^31,32^, where empty trap defects are slowly filled with charge carriers by radiation; then the dose or time of exposure can be determined by observing the number of photons produced during a luminescence measurement. The traps are extremely stable and stay populated for thousands of years. This process can be reversed to make excellent thermal history monitors^27,33,34^. In this technique, one chooses TL or OSL materials with a series or distribution of traps in the bandgap. These traps are filled before the measurement using ionizing radiation in the form of ultraviolet (UV) light, gamma rays, X-rays, neutrons, or beta particles (UV in many cases being the easiest to use). Particles of the materials can then be placed in a thermal event, such as an explosion. The shallow traps will empty preferentially over deeper traps, leaving a skewed population. Examination of the luminescence of the particles is literally as simple as sweeping up some of the explosion debris and heating it steadily while measuring light intensity. The measurements can be performed right away or many months or years after the explosion. The presence or absence of extraneous non-luminescent debris is irrelevant since the reconstruction of the thermal history depends on the ratios of the luminescent intensities (that is, populations) of the deep and shallow traps, not their absolute intensity. A conceptual diagram of the process is shown in Figure 1.

The simplest model for the rate of change of the population of a single trap (or species of traps) involves an Arrhenius expression:

(1)dndt=−nsexp[−EkT]

where *n* is the trap population density, *s* is a frequency factor for interactions with the trapped charge (for example, phonons), *E* is the trap energy depth, *k* is Boltzmann’s constant, and *T* is temperature. With some manipulation, we can extract an expression relating the population density versus time as a function of the thermal history of a particular event:

(2)n(t)=n0exp(−∫0tsexp(−EkT(t)))

**Figure 2:**
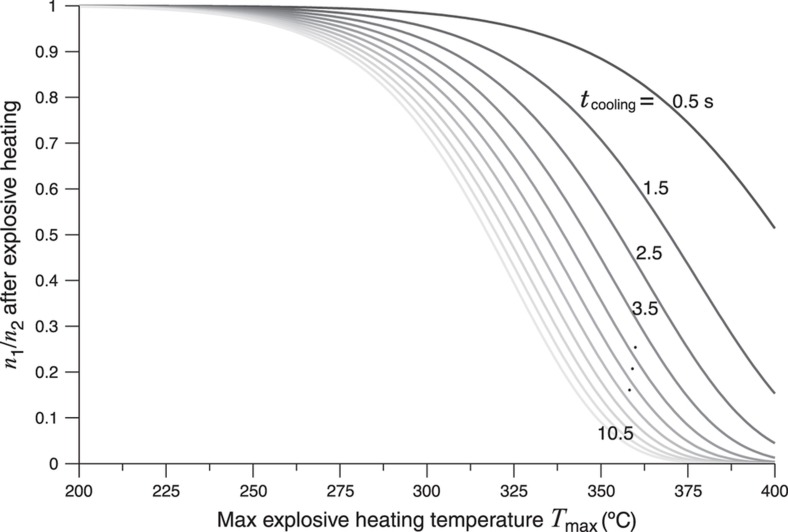
Ratio of the populations of a shallow trap (1) and deep trap (2) after a quick rise to a maximum temperature and then a slow cool. Note that the population of the shallow trap empties over a small temperature range even when the cooling time varies by an order of magnitude^27^.

**Figure 3:**
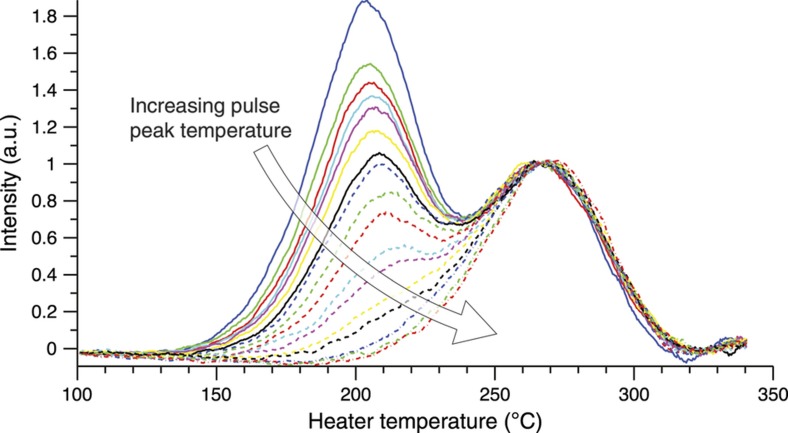
Thermoluminescent intensity versus temperature (‘glow curve’) for an Mg_2_SiO_4_ particle after being subjected by microheater to ~190 ms heating pulses of varying maximum temperatures. (An SEM of a similar particle and microheater can be seen in Figure 11.) Note the two peaks, each corresponding to a different class of trap^34^.

**Figure 4:**
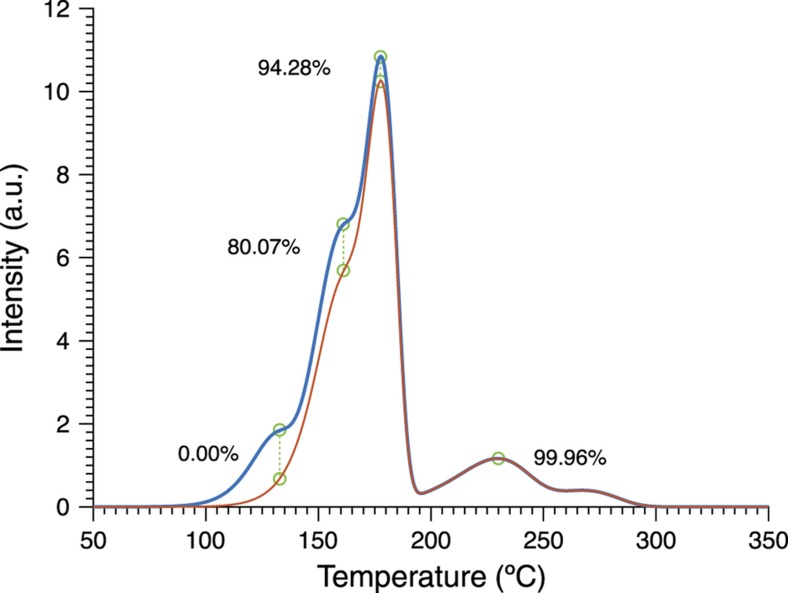
Simulated thermoluminescence of an LiF:Mg,Ti material before (blue) and after (red) being subjected to a detonation-type thermal event. The green lines and circles show the comparison points used in reconstruction.

The trap contributes two factors: trap depth and frequency factor. The first is clearly critical as an exponential term, but the frequency factor also gives another subtle dependence that varies between each class of trap. An analysis of population versus temperature and time is shown in Figure 2 assuming a temperature profile that begins at a maximum temperature and then cools with a slow exponential decay to room temperature. One can see that both the maximum temperature and duration of the cooling can alter the trap population, although clearly the temperature impact is strongest. By using multiple classes of traps, as shown by the TL intensity versus temperature plot (commonly referred to as a ‘glow curve’) in Figure 3, one has a sufficient number of degrees of freedom (in *E* and *s*) to reconstruct a specific time and temperature history for a simple thermal history curve. It should also be noted that multiple materials each with a single trap, for example, multiple particles or a multilayer, can be used as well for this purpose. The effect of a thermal event on the luminescence of a simulated TL material is illustrated in Figure 4. Many experimental tests involving the exposure of TL particles to explosives, combustibles, and microheaters have also been performed^24–28^.

### Irradiation and trap filling

TL is mostly the result of trapping and recombination of charge carriers occurring on an imperfect crystalline insulator or semiconductor. When an insulating crystal is exposed to ionizing radiation, populations of free electrons and holes are produced respectively in the conduction band and in the valence band of the material. In a perfect crystal, these charge carriers can recombine almost immediately, leading to prompt emission of light. In an imperfect crystal, however, namely a crystal containing defects that break the crystal periodicity (for example, vacancies, interstitial atoms, substituted atoms and so on), localized energy levels are introduced within the crystal’s energy bandgap. These energy levels can localize (capture) charge carriers (electrons and holes) created by ionizing radiation, effectively ‘storing’ the charge until a later time when the crystals can be readout. (Because of the large—often >6 eV—bandgap in insulating materials, deep UV is required to create electron–hole pairs sufficient to ionize specific defects in the crystal.)

The amount of information stored depends naturally on the concentration of available ‘trapping centers’, as well as on the dose of radiation to which the material has been exposed. For a given material, the amount of trapped charge typically increases with the dose of radiation (energy deposited per unit mass).

### Recombination and emission characteristics

**Figure 5:**
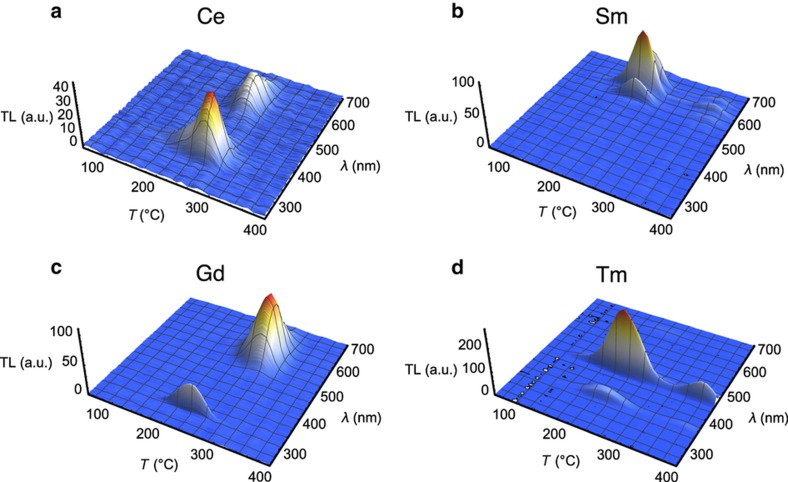
TL emission spectra of MgB_4_O_7_ doped with (**a**) Ce, (**b**) Sm, (**c**) Gd, and (**d**) Tm and co-doped with 1% Li^35^. TL, thermoluminescence.

Recombination of detrapped carriers can take place directly between conduction and valence bands, or with the assistance of a dopant or defect acting as a recombination center. The active process essentially determines the wavelength(s) of light associated with the TL emission. This can be clearly exemplified by examining the TL emission measured as a function of both temperature and wavelength for materials doped with different lanthanides. Figure 5 illustrates that for MgB_4_O_7_, where one can see that the type of dopant determines the TL emission wavelength. In this case one can observe the Ce^3+^ emission at ~340–460 nm, an intrinsic emission at ~550 nm, various Sm^3+^ lines in the 560, 600, and 645 nm, Gd^3+^ emission at 312 nm, and Tm^3+^ emission at 355, 455, and 475 nm. The ~550 nm emission in the Gd-doped sample is related to Mn contamination^35^.

Luminescence centers in which the electrons are coupled with the lattice vibrations are characterized by broad bands (for example, Ce^3+^) and their luminescence efficiency decreases with temperature, a process called thermal quenching. This decreases the intensity of high-temperature TL peaks. The lanthanide luminescence centers characterized by emission lines (for example, Sm^3+^, Gd^3+^, Tm^3+^) are associated with transitions between the 4*f* shells, which are shielded from the surroundings by filled orbitals (5*s*^2^ and 5*p*^6^), and do not suffer from thermal quenching^36^.

Thermal quenching does not represent a problem *per se* in temperature thermometry using TL, because the TL curve is measured in laboratory and one looks at how much the temperature changes the intensity of the TL curves—in other words, the technique relies on a relative comparison. Thermal quenching affects in the same way both the TL from the control samples and from the samples previously exposed to a temperature profile. Nevertheless, to develop materials with TL peaks over a wide range of temperatures, it can be useful to avoid thermal quenching by using lanthanides associated with *f–f* transitions.

Knowledge of the emission spectra of the TL materials is important both to understand fundamental TL mechanisms and, from a practical viewpoint, to select the optical filters used to measure the TL curve. Materials emitting with short wavelengths are also favored for TL measurements at high temperature, because short-wavelength emissions are easier to discriminate from the long-wavelength, non-signal blackbody background that starts to dominate the TL curves at high temperatures, depending on the materials and optical filters used.

### Alternative trap models

To date, most of the tests have utilized materials such as TLD-100 that follow^37^ the simple trap behavior described above. This is known as the first-order or Randall–Wilkins model, considered the foundational description of TL^38^. The exponential profile heavily weights temperature with respect to time, which fits well with many activation and accelerated aging processes^39^. The underlying assumption behind this model is that a short exposure to high temperature gives the same thermal dose as longer exposure to low temperatures, with the exact quantitative weight being given by the Arrhenius expression. However, there are thermal processes where this weighting may not be optimal. A good example would be a thermal treatment to remove solvent from a polymer mixture, such as baking photoresist after lithography^40^. In this type of process, a long exposure to low temperatures (for example, ~150 °C) is necessary to get the necessary reduction in solvent concentration, and a short exposure to very high temperatures will do nothing but damage the polymer.

If a thermal history model were to assess such a process, it would need both temperature- and time-dependent components, but some aspect of the time component should be linear or at least approximately so. Fortunately, there is a very common trap process that leads to a suitable time model: tunneling. There are a huge variety of tunneling processes, and many of them have rather minimal temperature dependences. Examples of these are direct tunneling^41^ and Fowler–Nordheim tunneling^42^. Further, there are a variety of TL and OSL materials with trap characteristics that indicate tunneling-assisted detrapping^43,44^. Indeed, the entire topic of anomalous fading^45–47^ in these materials is understood to be a tunneling process competing with traditional thermal detrapping. Using two specially chosen materials in a bilayer or multiple particle thermal history device, one could incorporate a tunneling process and an Arrhenius process simultaneously in a specific heat treatment, allowing one to assess temperature and time with very different weighting models. It is unlikely that the two materials would assess temperature and time completely independently, but with such strongly divergent weightings given by the underlying physics a very good thermal history reconstruction could be expected.

With this philosophy in mind, it would be useful to review some of the processes identified in TL and OSL materials that would lead to non-Arrhenius models. Many times these behaviors are considered undesirable, so we are in the fortunate situation of possibly being able to apply ‘rejects’ from the TL and OSL communities to a new application. As mentioned above, a number of materials show tunneling behavior. The most well-known class of these display ‘anomalous fading’, a process where a trap that, according to its thermal characteristics, should have a lifetime of thousands of years, unexpectedly depopulates over time. This is explained very successfully as a tunneling process^48,49^. In addition, de Lima *et al*.^43^ have modeled calcite as having luminescent traps with thermally-assisted tunneling, where the tunnel emission occurs from thermally-excited states of the trap. This concept of excited state tunneling in luminescent materials has also been used to model neighbor interactions between defects^44^. Tunneling has also been utilized to explain a 1/time dependence of decay in phosphor materials^50^.

In addition to tunneling processes, there are a number of variations in luminescence intensity besides the strict simple trap of the first-order model. The simple trap model assumes that an electron (or hole) in a filled trap is thermally excited to the conduction (or valence) band and then travels to a luminescence center and recombines. However, there is a finite probability that the carrier in the band will retrap before recombining. When this retrapping process is significant, the depopulation of carriers with temperature is slower and follows what is called a second-order model^48^, where the luminescence decays more slowly with increasing temperature than a first-order model would predict. There are also other higher-, general-, and mixed- order models to describe other variations in TL glow curves, as well as a push to replace the entire order-of-kinetics model^51–54^. Other models include a semi-localized transition model^55^ which has been invoked to explain non-physical frequency factors, where the factor *s* from Equation (1) has values far beyond any possible phonon interaction frequency, and anomalous heating rate effects, where the number of photons emitted increases with heating rate. There are also add-on effects such as the previously mentioned thermal quenching effect in some materials such as Al_2_O_3_:C (Ref. 56) where the number of photons emitted decreases with heating rate.

It is impossible to discuss every model that has been developed over the decades to explore every aspect of luminescent emission, but this short review merely points out the wide variety of luminescent behavior versus temperature and time; a more comprehensive tour can be had in the excellent work by Chen and McKeever^57^. For any given process, there will be many phosphor behaviors that can be chosen to track the most important features.

## Luminescent materials for temperature sensing

Based on the above discussion, the main requirements for materials to be used for temperature sensing can be summarized as:

- High TL intensity is desirable because of the interest in measuring the TL signal from single grains or small aliquots.
- Multiple TL peaks distributed over a wide range of temperatures broaden the applicability of the materials.
- Short-wavelength TL emission facilitates the discrimination between the TL signal and the background blackbody signal.
- Signal stability or absence of anomalous fading means that the decrease in the TL signal can be attributed to the temperature exposure of the particles prior to the readout. Normal signal decay due to the thermal fading imposes a lower temperature limit of measurement, which depends on the delay between particle preparation (irradiation) and readout.
- Light insensitivity is important to be able to handle the samples in light and avoid the interference of light exposure during the detonation event. If the material is not light-insensitive, temperature measurements are restricted to environments where exposure to light—including any short-wavelength radiation produced by the thermal event—can be minimized.
- Simple TL kinetics allows us to assume superposition of the TL peaks in the temperature reconstruction algorithms. Non-first-order kinetics does not preclude the use of TL particles as temperature sensors, but complicates the analysis.

Ideal materials that satisfy every requirement above are, of course, difficult to find. On the other hand, many of the requirements in TL dosimetry, such as low effective atomic number, do not apply for temperature sensing.

### Standard TL materials and light sensitivity

A variety of TL materials are available for TL dosimetry, the most important host crystals being LiF, CaF_2_, Al_2_O_3_, Li_2_B_4_O_7_, and CaSO_4_ (Ref. 58). These hosts materials are also available in a range of dopant formulations, such as LiF:Mg,Ti, LiF:Mg,Cu,P, CaF_2_:Dy, and CaF_2_:Mn, which determine not only their emission spectra but also TL properties such as sensitivity, position of the TL peaks, fading, and light sensitivity.

A survey of TL materials available for TL dosimetry shows that most of them are light sensitive, the clearest example being the highly radiation sensitive Al_2_O_3_:C (Ref. 59). This is not necessarily a problem for dosimetry, since the detectors can be properly packaged for light protection, but this is not an option in temperature-sensing applications.

Other TL materials show degradation of the TL sensitivity when the sample is heated above a certain temperature. LiF:Mg,Cu,P, for example, is a highly sensitive material, but its sensitivity decreases when the material is heated above 240 °C (Ref. 58). This clearly prevents the material from being used for temperature-sensing applications using the approach described here.

### LiF:Mg,Ti

LiF:Mg,Ti is arguably the most-used and most investigated material in TL dosimetry. The main advantage of this material is its commercial availability and the fact the TL peaks are relatively light insensitive^58^. Its TL curve consists of multiple peaks from −160 to 400 °C, dominated in dosimetry applications by peak 5 just above 200 °C (Refs. 60,61). The signal is relatively stable, with fading usually cited as less than 10% in a month^62^.

The TL curve can be fitted with first-order TL peaks, although the underlying physical model is probably more complicated than that described by simple charge trafficking between the localized energy levels and the delocalized energy bands. More recent models propose localized recombination between trapped charges within defect clusters. Supralinearity at high irradiation doses is also a well-known quirk.

The relative intensity of the TL peaks is determined by an equilibrium between defect complexes. It is not clear at the moment the effect of sudden temperature exposure to these defect complexes.

### MBO, LBO, and CSO

**Figure 6:**
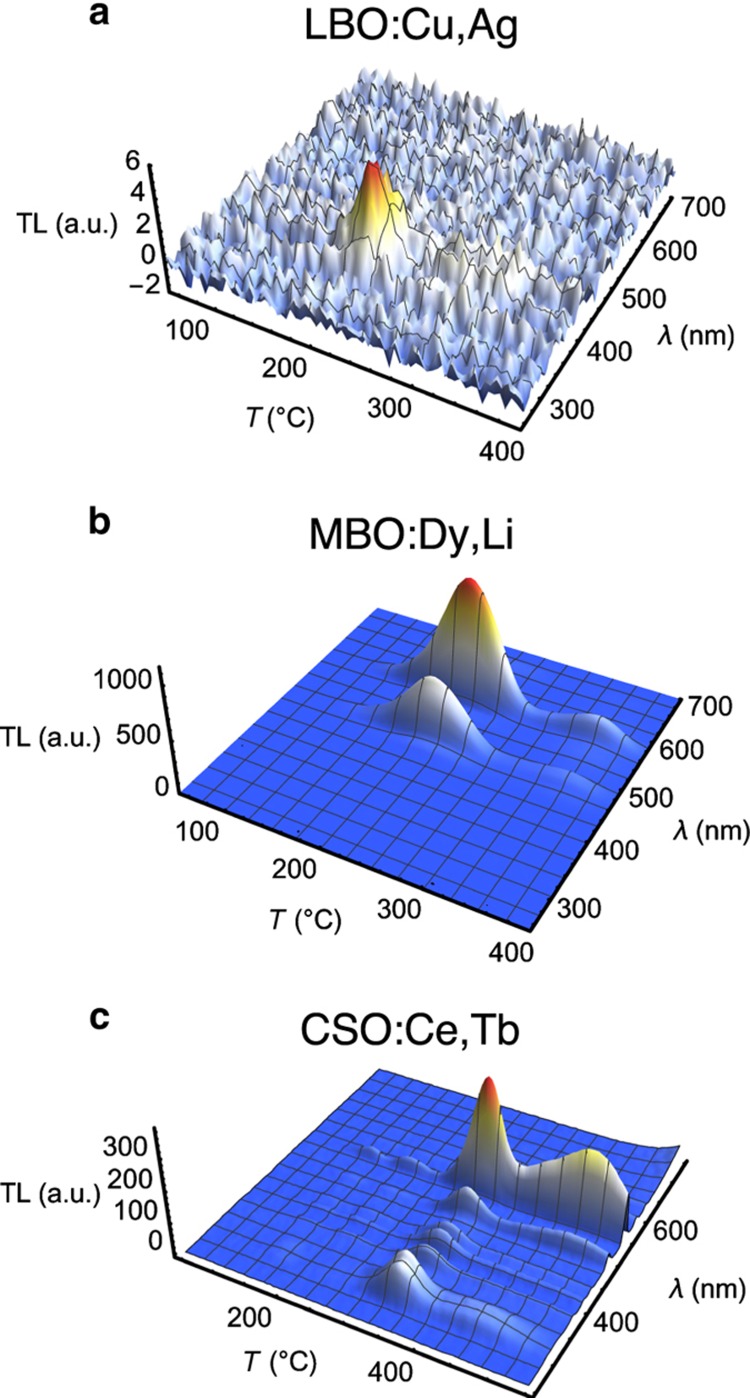
TL emission spectra of (**a**) LBO:Cu, Ag, (**b**) MBO:Dy, Li, and (**c**) CSO:Ce, Tb^64^. TL, thermoluminescence.

Because of the limited availability of TL materials for temperature-sensing applications, a targeted effort has been carried out to develop new materials specific for this type of application. The biggest challenge was to identify and develop materials that did not show light sensitivity, as in most materials light will lead to emptying of the electron traps and loss of TL signal. After a survey of multiple hosts and dopant combinations^63^, three hosts were identified as providing the least light sensitivity and, at the same time, TL properties that can be to a certain extent controlled by the choice of dopants. These host materials are Li_2_B_4_O_7_ (LBO), MgB_4_O_7_ (MBO), and CaSO_4_ (CSO); their TL emission spectra with a few example dopants are shown in Figure 6. Although LBO showed a weaker TL intensity than MBO or CSO, we include it here because the TL was light insensitive.

The synthesis and the main properties of these materials have been described by Doull *et al.*^64^, with partial investigations on the effect of synthesis conditions described by others^35,65,66^. These materials have also been subjected to laboratory and closed-chamber tests^67,68^.

### Multilayer materials: Thermal history as a function of depth

In addition to the physical behavior and modeling of the luminescence process, one can design the geometry of materials to better extract thermal history. For example, consider a thermoluminescent bilayer structure that is exposed to a rapid initial period of high temperature and then a lower subsequent temperature. The bilayer will have a surface layer that directly faces a thermal process and a deeper layer that sits behind it. In the initial high-temperature period, the surface layer will feel the full effect of the high temperature, but the deeper layer will only see the effect of the temperature as it diffuses through the surface layer. This means that the surface will end up sampling the intense initial temperature pulse while the deeper layer samples a damped thermal average over a longer period. If the layers are chosen properly, then their luminescent emission will occur at different wavelengths, and both layers can be independently measured simultaneously. This concept can be extended to large multilayers or core-shell particles, where each layer measures temperature over a different timescale. In this manner, one can design thermal dose monitors to measure the temperatures of multi-step processes, where one or more later steps occur at temperatures lower than the initial ones. It is interesting to note that this process is very similar in principle to one used in the earth sciences where temperature readings, for example, from a cave^69^ or an ice core^70^, which are taken from the surface to great depth, can be analyzed to reconstruct an average temperature over time back to thousands or hundreds of thousands of years.

**Figure 7:**
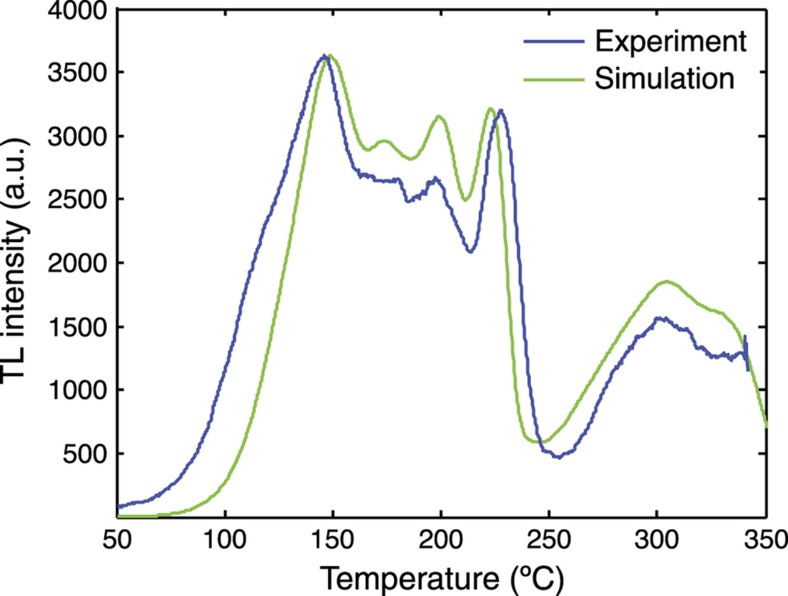
Experimental and simulated thermoluminescence versus temperature for a bilayer of material that is heated from one side. The depth into the material indicates thermal interval^71^.

Kim *et al.*^71^ have performed an experiment that demonstrates this principle. In that work an adhesive-bonded bilayer of CaF_2_:Dy and LiF:Mg,Ti dosimetry chips was constructed, and the top surface of the CaF_2_:Dy was coated with a thin aluminum layer. This bilayer was then irradiated with X-rays to fill the traps of the two luminescent materials. The bilayer was exposed to pulses of Nd:YAG laser light at *λ*~1064 nm that heated the sample from the absorption of the aluminum. These pulses had durations on the order of hundreds of milliseconds and were varied to obtain different levels of heating. It was found that they could explain the luminescence characteristics of the bilayer by modeling the heat transfer through the bilayer as a diffusion process with appropriate interface contributions to the thermal contact conductance. Figure 7 shows a luminescence curve of this type, showing the combined emission (both experimental and simulated) of CaF_2_:Dy and LiF:Mg,Ti after exposure to a 500-ms laser pulse. CaF_2_:Dy is in general a much brighter phosphor than LiF:Mg,Ti when integrated over the entire spectrum, and one would expect to have difficulty seeing the contribution of LiF:Mg,Ti, but by monitoring the luminescence at a specific wavelength where the two materials have comparable intensities this problem is easily overcome.

This concept of using the depth inside a material as a proxy for time has potentially exciting applications, but it should be noted that is has limitations as well that are dictated by size. Time scales longer than a few seconds are difficult to implement because the device size becomes unwieldy. Also there are problems for very small sizes. Core-shell nanoparticles are not very effective in measuring heating over common time scales. The thermal diffusion time of a sub-micron nanoparticle can be on the order of a nanosecond or less, and so such a particle will not have a physically meaningful temperature difference between its surface and core layers during any process that lasts longer than this. (Just to avoid confusion, we should reiterate that this section is explicitly considering only the use of multilayers where different depths into the multilayer represent different intervals of time. The normal use of multilayers as a source of different luminescent materials or peaks is always valid even for nanoparticles.) However, these limitations aside, when a process requires a temperature step that lasts for many nanoseconds to seconds, the use of multilayer or core-shell materials gives a hitherto unexplored means of extracting temperature versus time.

## Thermal history reconstruction techniques

The impact of a thermal event on the carrier population density *n*(*t*′) of a trap with depth *E*, frequency factor *s*, and initial population *n*_0_ was described in a prior section. If a glow curve is subsequently collected from a trap species so affected, the intensity seen is predicted by the Randall–Wilkins first-order kinetics model to be

$$\begin{array}{c} I\left(t\prime \right)=C\frac{dn}{dt}\\ =C{n\prime }_{0}\;\exp\left(-\int_{0}^{t\prime }se^{-E/kT\prime (\tau \prime )}d\tau \prime \right)se^{-E/kT\prime (\tau \prime )} \end{array}$$

where ${n\prime }_{0}$ is the carrier density after the initial thermal event and *T*(*t*′) is the linear heating applied to collect the glow curve^72^. A TL material which conforms to this model can be viewed as a collection of independent trap species, with their TL curve intensities summing to produce the overall material curve.

**Figure 8:**
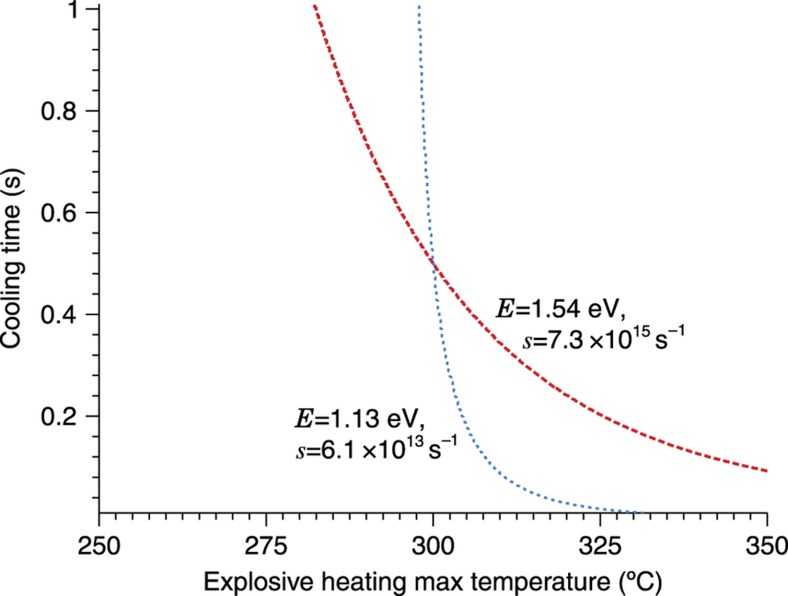
Two simulated first-order traps, explosively heated to a certain maximum temperature and then allowed to cool with a certain time constant, will each display a certain final population density. Each trap could also reach the same population density via a series of slightly different scenarios, which can be represented on the plane defined by the two heating parameters as a line of equivalent depopulation; however, only one scenario—located at the intersection of these lines—will match both traps with the ‘actual’ population densities that the post-detonation glow curve would indicate. The task of thermal history reconstruction is to deduce information from these lines of equivalent depopulation and find the heating scenario where all traps (ideally) agree^26^.

**Figure 9:**
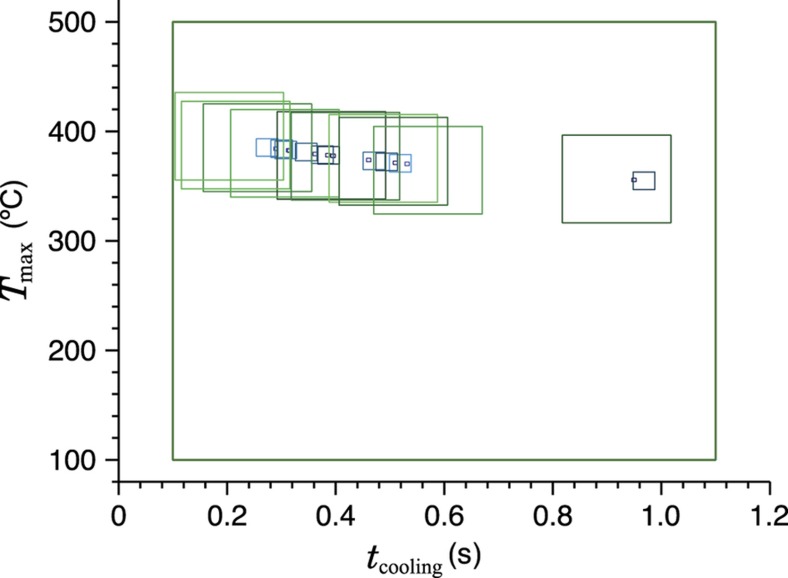
An illustration of an iterative search process reconstructing the two-parameter explosive heating history. The two-dimensional plane of possible heating scenarios is sampled at uniformly distributed points and the quality of the match between each point’s simulated glow curve and the actual observed TL calculated. A certain number of more tightly-bounded planes are established, centered at the points which most closely matched the data, and the process repeats in each of these planes. Observed in graphical form, progressively smaller boxes highlight the search areas of each iteration and outline the shape of the lines of equivalent depopulation. Four iterations of area refinement are visible here. (The simulated material contains several traps, but due to their parameters and the particular scenario being sought the equivalent depopulation lines are fairly close together^26^.) TL, thermoluminescence.

**Figure 10:**
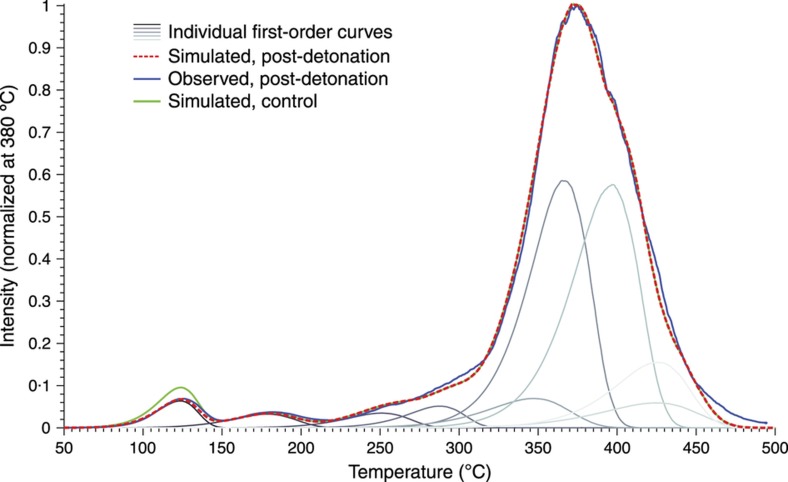
The glow curve of a sample of CaSO_4_:Ce,Pr particles retrieved after experiencing the detonation of a 5 lb pentolite charge in an open outdoor test structure, accompanied by simulated glow curves before and after undergoing the 220 ºC, 0.20-s thermal event found to most closely match experimental results. Note that unlike LiF:Mg,Ti, CaSO_4_:Ce,Pr has not been widely confirmed to behave as a handful of first-order traps, but the reconstruction concept is completely compatible with other physical TL models. TL, thermoluminescence.

For use as a thermal history sensor, we wish to reconstruct the unknown thermal history *T*(*t*) by starting from the final TL curve intensity *I*(*t*′). Unfortunately, while numerically evaluating the intensity expression to simulate a curve is easily accomplished, the reverse process of solving directly for *T*(*t*) is generally intractable. We therefore begin by assuming a functional form for the thermal history that can be described using a handful of parameters; this solution space is then canvassed for the combination of thermal parameters which results in a simulated intensity versus temperature curve closest to the actual observed. The ‘correct’ scenario, where each individual trap species amount of depopulation and consequent TL intensity decrease, is in theory unique as long as there are more trap species than thermal parameters in the (non-symmetric) scenario and the trap parameters are sufficiently separated, as shown in Figure 8, although experimental error usually interferes in real materials. Performance is enhanced by using some form of iterative search or optimization algorithm, as illustrated in Figure 9. While this approach depends on the correct form of thermal event being known and describable by a small number of parameters, it is robust and adaptable, compatible with different thermal event profiles and any TL model capable of numerically generating *I*(*t*′) from *T*(*t*). Figure 10 gives an example using experimental data.

## Case study: measuring thermal history inside explosions

### Laboratory testing: Microheaters

Before one tries to directly measure the temperatures in an explosion, it is imperative to be able to test TL particles using a low cost and lab-friendly technique that can reproduce very rapid (milliseconds) temperature increases of hundreds of degree Celsius with arbitrarily slow cool-down times. Micromachined heaters provide an excellent means to do this since their temperature can be controlled by applying a small time-varying or steady-state current, and the small size of the heater means they can heat and cool at millisecond-scale rates. This section describes the characteristics and use of these heaters.

**Figure 11:**
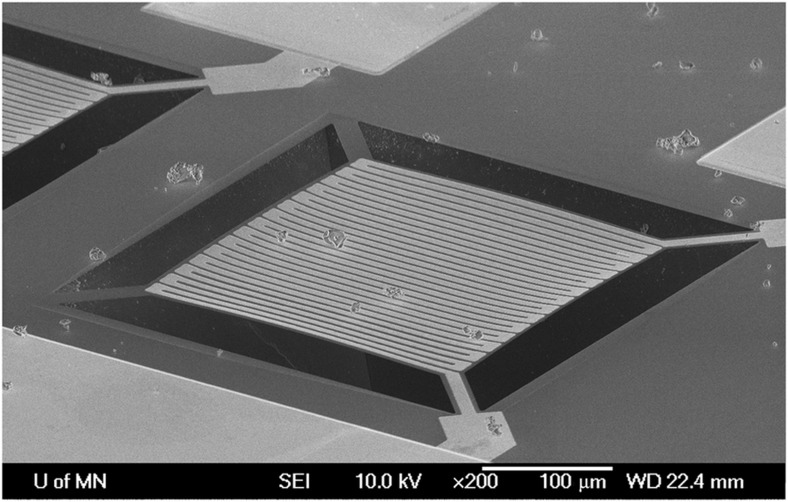
An SEM image of a 300 μm by 300 μm microheater, dotted with microparticles of the TL material Mg_2_SiO_4_. The heater comprises a 160 nm Pt resistor on a 200 nm LPCVD low-stress silicon nitride platform; a cap layer of PECVD nitride protects the resistor’s Cr adhesion layer from attack during the KOH platform release etch. The scuffing visible on the gold contact pad at bottom is from the use of a microprobe to make electrical contact during testing^24^. TL, thermoluminescence.

TL material readout is conventionally done on a hotplate or planchet heater, heated by driving a large electrical current through a resistive material. While this generates heat well-enough, the large thermal mass of these devices makes them ponderous to heat and cool and difficult to precisely measure and control^73^. Many of these problems can be alleviated by miniaturizing a resistive heater using VLSI microfabrication methods. A serpentine resistor and an enclosing platform, both hundreds of nanometers thick, are essentially the only components in the body of the resulting ‘microheater’, an example of which is pictured in Figure 11. Etch-releasing the platform from the substrate drastically limits the thermal conduction path to bulk material, and contributions from radiation, air conduction, and convection are typically much reduced at microscale; this high isolation and low thermal mass reduces the heating time constant to the order of tens of milliseconds^74^. The microheater’s temperature can be accurately measured using the drive resistor’s thermal coefficient of resistance, highly linear and thus easily calibrated in materials such as platinum^75^. Designs may also be tailored for unique capabilities, such as higher-temperature resistors^76^, a low-emittance platform material with resistors moved to the periphery to reduce emissivity^77^, or the addition of a multilayer optical interference filters to dampen certain wavelengths of blackbody radiation^78^. With many designs, the high-thermal isolation, low-response times, and built-in sensing simplify thermal analysis such that closed-loop control schemes are not necessary for acceptable precision.

**Figure 12:**
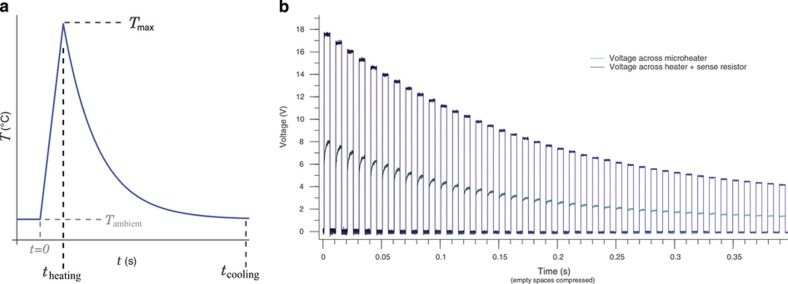
A series of temperature pulses, shown in (**b**), administered by microheater recreates the temperature profile (**a**) of a post-detonation environment: rapid heating followed by exponential cooling. Each pulse is driven by a voltage (top curve) administered for 5 ms by a programmable pulse generator; a power resistor in series with the microheater allows monitoring (bottom curve) of the heater’s resistance, and thus temperature, via digital oscilloscope. The time between pulses has been truncated in (**b**) for display.

The greatly increased heating speed conferred by a microheater is useful for more than standard glow curves. Precisely-clipped temperature ramps are key to the thermal cleaning and fractional glow techniques for probing trap structure^48^. Microheaters can provide rapid temperature excursions of several hundred degrees Celsius lasting under a hundred milliseconds, which is on the order of those found in a post-detonation environment. An arbitrarily complex temperature profile can be driven by a simple square-wave pulse generator if it is administered as a series of separate temperature pulses, as illustrated in Figure 12, which will provoke a theoretically identical response in a first-order TL material. As seen with Mg_2_SiO_4_ in Figure 3, microparticles (or directly-deposited films) of TL materials can be irradiated, pulse-heated to imitate a detonation, characterized with a glow curve, and the entire process repeated, all without breaking contact from the microheater^24^.

The advantages of pushing heaters into the microscale regime do not come without new complications. Mistakes in design or fabrication could result in non-uniform heating across the platform, which becomes a significant problem in fast operation; residual stress and thermal expansion differences in the device’s layers can cause platforms with pronounced curvatures. (The shape can also be bistable with a temperature-triggered transition, resulting in microheaters which abruptly catapult their cargo away when they reach a certain temperature.) Stiction forces are quite capable of keeping smaller microparticles adhered in place, but movement in response to mechanical shocks becomes a risk with particles larger than ~100 μm. Even with a stably flat microheater, variations in thermal contact at micrometer dimensions and millisecond heating times can have a significant effect on the temperature that an irregularly-shaped particle will actually reach. Monitoring of the microheater’s realized resistance for slope or other discontinuities, consideration of departures from calibration results, and post-experiment visual examination to confirm that the samples have not moved are necessary habits.

### Particle response to explosions

While microheaters and furnaces can provide a laboratory-safe simulation of explosive detonations, the detonation environment itself provides many more challenges to a sensor’s survival than just temperature excursions. Shock waves of thousands of psi, gigapascal pressures, highly energetic chemical reactions, and violent physical collisions with debris await in and around a detonation fireball^79^. Most of these effects can be feasibly reproduced one-at-a-time in the laboratory, but a prospective sensor must be resistant to all of these effects in combination.

The most accurate test to measure a thermoluminescent particle’s response is to subject it to an actual explosion. In our work, TL microparticles have been tested numerous times under a variety of explosion conditions, but we will define three general types that overlap somewhat but to which we will refer in the discussion below: survivability testing, closed-chamber testing, and open chamber testing. Survivability refers to testing that is only meant to determine if the particles survive an explosion with their properties intact. It is not meant to measure temperature. These types of tests can be closed or open chamber. Closed-chamber testing occurs in a heavily reinforced container in which a small explosive charge is placed and detonated. The container generally induces an approximately uniform set of conditions during the test due to its small volume, which means that TL particles often measure similar (but not identical) temperatures within the chamber. An open chamber test occurs in a structure with one or more openings to the atmosphere where overpressure can exit from the system during the blast. Temperature measuring particles are free to follow the expanding fireball, the intense turbulent mixing within the structure, and outflow from that structure.

**Figure 13:**
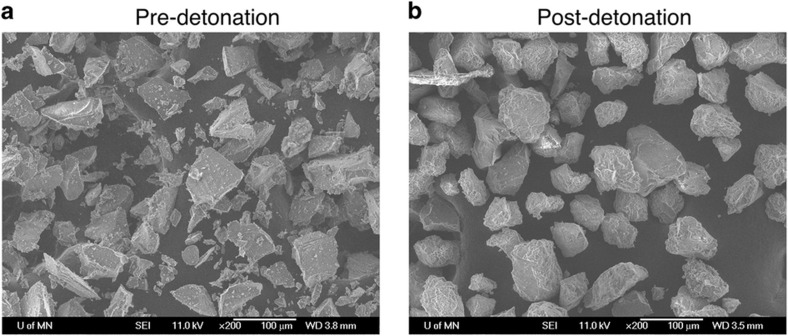
SEM images of Al_2_O_3_:C particles before (**a**) and after (**b**) experiencing a 25 g PETN charge detonation at very close range (~1ʺ or less). Although some changes in shape and surface chemistry were noted, no alteration in TL characteristics could be discerned. In both cases, the particles are imaged while adhered to carbon tape^27^. TL, thermoluminescence.

**Figure 14:**
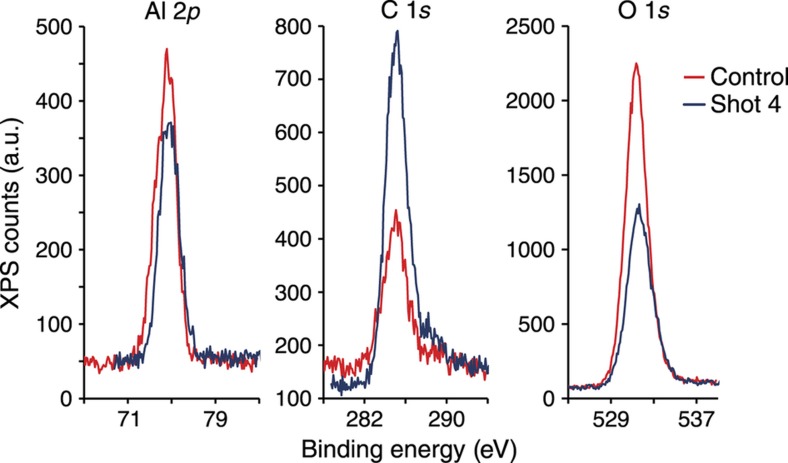
XPS spectra of Al_2_O_3_:C, centered on the primary peaks of its elemental constituents, before and after detonation of a 25 g PETN charge^27^. XPS, X-ray photoelectron spectroscopy.

A series of tests performed with collaborators at the Naval Surface Warfare Center’s Indian Head Division measured the survivability of several common TL materials in closed-chamber detonations. The initial test material used 15–90 μm particles of Al_2_O_3_:C, which displays a single strong TL glow peak when irradiated with broad-spectrum UV light. A high-explosive charge, comprising approximately either 2 or 25 g of PETN and a detonator, was immersed in a paper cup of Al_2_O_3_:C particles and then triggered. Samples were recovered from a plastic collection vessel situated below the detonation package. SEM imaging of the collected particles, as shown in Figure 13, revealed that the Al_2_O_3_:C, originally angular bodies with sharp corners and smooth planes, have been reshaped into much more rounded, compact configurations with high-surface roughness. Qualitative observation also noted a relative dearth of recovered particles at the smaller end of the size range (below about 25 μm), but this could easily be a side effect of the handling and collection processes. Chemical analysis using X-ray photoelectron spectroscopy, shown in Figure 14, indicated a relatively large decrease in the aluminum present on the post-detonation particle surfaces; coupled with a smaller dip in oxygen content, this suggests the combustion of atomic aluminum. A small increase in carbon is also observed, but could stem from combustion products deposited on the particle surfaces.

**Figure 15:**
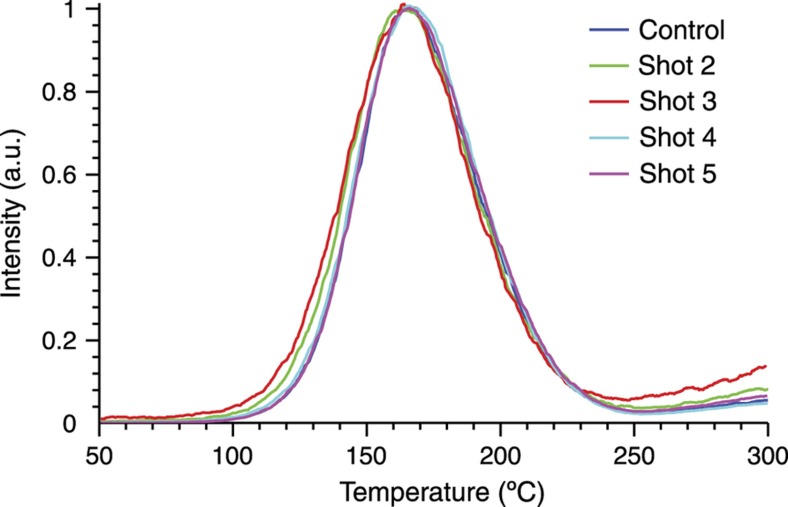
Thermoluminescence glow curves of four sets of Al_2_O_3_:C particles exposed before irradiation to PETN detonations, compared with a pristine control. All glow curves were stimulated using a linear heating ramp of ~0.9 °C s^−1^ (Ref. 27).

Most importantly, TL glow curves were collected from the detonated Al_2_O_3_:C. (As the goal was to detect changes in the thermoluminescent properties, rather than attempt temperature reconstruction, the samples were not UV irradiated until after detonation.) As evident in Figure 15, all post-detonation samples exhibited glow curves that were visually indistinguishable from that of the control particles, with peak width (FWHM) and locations varying by less than the experimental readout error. The constancy of the glow curves suggests that the physical and chemical changes observed do not alter the material’s basic TL functionality. This durability has been upheld by other materials, such as TLD-100 and doped calcium sulfate, used in subsequent detonation tests.

Several closed-chamber tests have been performed at the Indian Head Naval Surface Warfare Center in Maryland, and multiple open chamber tests have been performed at Kirtland Air Force Base in Albuquerque, NM. Before discussing some example data, it is worth re-emphasizing that sample collection after the explosion requires no special technology or procedure. One merely brushes some of the dust or debris from an area of interest into a small vial. Such an amount will be sufficient for many measurements because in many cases even a single TL particle will be sufficient to extract a temperature, although usually a larger amount is used.

**Figure 16:**
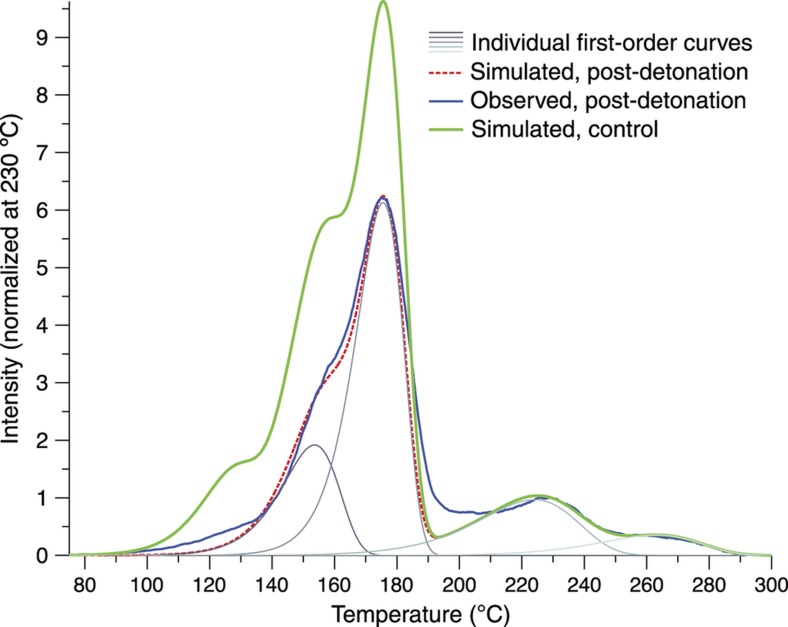
The thermoluminescence signal of a sample of TLD-100 particles after a closed-chamber detonation is compared with several simulated glow curves: a pre-detonation curve, modeled from a control sample; a simulated curve after experiencing a detonation-style thermal event reaching a maximum temperature of 240 °C and cooled in about 0.4 s; and the individual first-order trap species which are summed to form the material model, most of which are calculated to be heavily depopulated here after the detonation. Modified from Ref. 26.

The TL data of a sample of debris containing LiF:Ti,Mg particles are shown in Figure 16. This glow curve was taken after the closed-chamber detonation of 20 g of aluminized HMX, initially situated about 22 cm from the LiF:Ti,Mg sample. From the reconstruction process described above, it was determined that the explosion had a peak temperature of ~240 °C with a cooling time of about 0.4 s. A thermocouple positioned at the same distance from the charge reported peak temperatures of ~210 °C with cooling times of a few seconds, which agrees well with the microparticle results given that the thermocouples are more massive and thus heat and cool more slowly^26^.

It is usually seen that temperatures from position to position in a closed-chamber test do not vary by more than a few tens of degrees Celsius. We have noted that in open chamber tests, large variations are far more typical. Several tests have been performed where open-top boats of CSO and MBO particles are suspended within a multi-chamber open structure for the detonation of a multi-pound Pentolite charge; particles began in positions varying from within 3 m to out of direct line of sight of the detonating charge, and were collected—along with sand, metal fragments, wood chips, and other debris—from predetermined ~1 ft^2^ collection sites. Some recovered particles were found nearly completely bleached of all TL signal, requiring an exposure temperature approaching several hundred to 1000 °C, while other particles collected at exactly the same site retain all of their TL, indicating minimal if any exposure to elevated temperatures. This implies that the particles are scattered in an environment where temperature fluctuations vary wildly with time and position. Every collection site seems to contain particles from a wide variety of thermal paths, although the average temperature from site to site varies quite strongly as well. These average temperatures taken over many particles match well with thermocouple data, which read maximum temperatures from 200 °C to 500 °C in varying locations. Temperature corrections using the overall sample luminescence must be performed when a significant fraction of the particles are bleached (at very high temperatures) since bleaching shifts the remaining TL distribution such that it indicates artificially low temperatures. Once this correction is made, good agreement with thermocouples is obtained.

## Conclusions

Thermoluminescent particles make highly successful microsensors to measure thermal history, particularly for—but not limited to—uniquely harsh environments such as the immediate proximity of a high-explosive detonation. The particles are hard durable oxides and fluorides that can withstand very harsh environments without contaminating their surroundings or decomposing. To date, they operate based on an Arrhenius expression, which emphasizes the highest temperatures that are most important in determining a thermal dose. The particles are small enough to be embedded in material or process and they are easily read without needing to be separated from their matrix. They are nonvolatile, can be activated using a variety of radiation sources many months before they are needed as thermal history sensors, and can be stored for many months after sensing before they are read. In the future, successful deployment of the technique will depend on a close collaboration between researchers with expertise in TL methods and materials, and end-users interested in employing thermal history sensing.
